# Supplementary material for: Context-Seq: CRISPR-Cas9 targeted nanopore sequencing for transmission dynamics of antimicrobial resistance
Source: Nat Commun. 2025 Jul 3;16:5898. doi: 10.1038/s41467-025-60491-0 (PMC12229574; doi:10.1038/s41467-025-60491-0)
Supplement: Supplementary file 2 — Description of Additional Supplementary Files [file 41467_2025_60491_MOESM2_ESM.pdf]

Description of Additional Supplementary Files

File Name: Supplementary Data 1
Description: Top five BLASTN taxonomy matches and Kraken2 taxonomy for consensus sequences.
