# Supplementary material for: Context-Seq: CRISPR-Cas9 targeted nanopore sequencing for transmission dynamics of antimicrobial resistance
Source: Nat Commun. 2025 Jul 3;16:5898. doi: 10.1038/s41467-025-60491-0 (PMC12229574; doi:10.1038/s41467-025-60491-0)
Supplement: Supplementary file 4 — Reporting Summary [file 41467_2025_60491_MOESM4_ESM.pdf]

nature portfolio

Corresponding author(s): Amy J. Pickering

Last updated by author(s): Sep 11, 2024

Reporting Summary

Nature Portfolio wishes to improve the reproducibility of the work that we publish. This form provides structure for consistency and transparency in reporting. For further information on Nature Portfolio policies, see our Editorial Policies and the Editorial Policy Checklist.

Statistics

For all statistical analyses, confirm that the following items are present in the figure legend, table legend, main text, or Methods section.

n/a	Confirmed	
☐	☒	The exact sample size (*n*) for each experimental group/condition, given as a discrete number and unit of measurement
☐	☒	A statement on whether measurements were taken from distinct samples or whether the same sample was measured repeatedly
☐	☒	The statistical test(s) used AND whether they are one- or two-sided<br>*Only common tests should be described solely by name; describe more complex techniques in the Methods section.*
☒	☐	A description of all covariates tested
☒	☐	A description of any assumptions or corrections, such as tests of normality and adjustment for multiple comparisons
☐	☒	A full description of the statistical parameters including central tendency (e.g. means) or other basic estimates (e.g. regression coefficient) AND variation (e.g. standard deviation) or associated estimates of uncertainty (e.g. confidence intervals)
☐	☒	For null hypothesis testing, the test statistic (e.g. *F*, *t*, *r*) with confidence intervals, effect sizes, degrees of freedom and *P* value noted<br>*Give P values as exact values whenever suitable.*
☒	☐	For Bayesian analysis, information on the choice of priors and Markov chain Monte Carlo settings
☒	☐	For hierarchical and complex designs, identification of the appropriate level for tests and full reporting of outcomes
☒	☐	Estimates of effect sizes (e.g. Cohen's *d*, Pearson's *r*), indicating how they were calculated

Our web collection on statistics for biologists contains articles on many of the points above.

Software and code

Policy information about availability of computer code

Data collection	We used the following open-source software for DNA sequence analysis: Guppy (v6.1.5, v6.3.9, v6.4.6, v6.5.7), Porechop (v0.2.4), Usearch (v11.0.667), racon (v1.4.20), medaka (0.11.5), Minimap2 (2.22-r1101), CARD (v3.2.6), mobileOG database (beatrix-1.6), Prokka (v1.14.5), Kraken2 (v2.0.7-beta), PlasX , BLASTn (2.12.0)
Data analysis	The analyses were conducted in R v4.3.2 using the following packages (all available through GitHub or CRAN): ggplot2, dplyr, reshape2, tidyverse, ggenes (0.5.1), genomicRanges (1.54.1), genomicAlignments (1.38.0). A custom guide design tool was developed and is available here: https://github.com/Shruteek/Optimized-sgRNA-Design)

For manuscripts utilizing custom algorithms or software that are central to the research but not yet described in published literature, software must be made available to editors and reviewers. We strongly encourage code deposition in a community repository (e.g. GitHub). See the Nature Portfolio guidelines for submitting code & software for further information.

Data

Policy information about availability of data

All manuscripts must include a data availability statement. This statement should provide the following information, where applicable:

- Accession codes, unique identifiers, or web links for publicly available datasets
- A description of any restrictions on data availability
- For clinical datasets or third party data, please ensure that the statement adheres to our policy

All raw sequence data were deposited in the NCBI database under BioProject accession number PRJNA1157857.

Research involving human participants, their data, or biological material

Policy information about studies with human participants or human data. See also policy information about sex, gender (identity/presentation), and sexual orientation and race, ethnicity and racism.

Reporting on sex and gender	This is not part of our analysis however reported sex of human samples are provided. The sex (based on reporting) for all sequenced human samples is available in supplementary table 2. Sample size was too small for disaggregated analyses.
Reporting on race, ethnicity, or other socially relevant groupings	This is not part of our analysis.
Population characteristics	We collected samples from poultry-owning households with at least one child under 5 years old in two subcounties in Nairobi, Dagoretti South (n = 25) and Kibera (n = 25). All households in Kibera used chlorinated piped water, while none of the water sources in Dagoretti South were chlorinated. Dagoretti South and Kibera had similar wealth indices, as measured by household assets like electricity, TV, mobile phones, and stoves. Poultry ownership in both areas was mainly for nutrient provision via meat and eggs or income generation. Over half of the respondents allowed poultry and canines into their homes, with study staff observing less animal feces near household soil sampling areas in Dagoretti South compared to Kibera.
Recruitment	Households were personally visited and selected for the study based on specific criteria: poultry ownership and the presence of at least one child under the age of 5 years. Eligible households were then invited to participate, with written informed consent obtained from each adult participant. For children, both child assent and parental written consent were obtained.
Ethics oversight	The study received ethical approval from the Kenya Medical Research Institute (KEMRI) Scientific and Ethics Review Unit (12/3823) and the Tufts Health Sciences Institutional Review Board (13205). Additionally, a research permit was granted by the Kenyan National Commission for Science, Technology, and Innovation.

Note that full information on the approval of the study protocol must also be provided in the manuscript.

Field-specific reporting

Please select the one below that is the best fit for your research. If you are not sure, read the appropriate sections before making your selection.

☒ Life sciences ☐ Behavioural & social sciences ☐ Ecological, evolutionary & environmental sciences

For a reference copy of the document with all sections, see nature.com/documents/nr-reporting-summary-flat.pdf

Life sciences study design

All studies must disclose on these points even when the disclosure is negative.

Sample size	No sample size calculation was conducted for this methodological study.
Data exclusions	No data were excluded from the analyses. Samples were selected based on the criteria listed in the manuscript.
Replication	We did not reproduce our analyses. All data required to reproduce our analyses are publicly available without restrictions.
Randomization	Urban informal settlements in Kenya are typically organized into compounds where multiple households share a common courtyard. In this cross-sectional study, one household was randomly selected and enrolled from each compound that owned poultry.
Blinding	No blinding was performed.

Reporting for specific materials, systems and methods

We require information from authors about some types of materials, experimental systems and methods used in many studies. Here, indicate whether each material, system or method listed is relevant to your study. If you are not sure if a list item applies to your research, read the appropriate section before selecting a response.

Materials & experimental systems

n/a	Involved in the study
☒	☐ Antibodies
☒	☐ Eukaryotic cell lines
☒	☐ Palaeontology and archaeology
☐	☒ Animals and other organisms
☒	☐ Clinical data
☒	☐ Dual use research of concern
☒	☐ Plants

Methods

n/a	Involved in the study
☒	☐ ChIP-seq
☒	☐ Flow cytometry
☒	☐ MRI-based neuroimaging

Animals and other research organisms

Policy information about studies involving animals; ARRIVE guidelines recommended for reporting animal research, and Sex and Gender in Research

Laboratory animals	The study did not involve laboratory animals.
Wild animals	The study did not involve wild animals.
Reporting on sex	Sex was not considered in the study design.
Field-collected samples	We collected poultry and canine fecal samples from each household. Field enumerators collected fresh fecal piles for poultry and canine samples which were kept in a cooler for transport to the lab. The collected samples were then aliquted and stored at -80C.
Ethics oversight	The study received ethical approval from the Kenya Medical Research Institute (KEMRI) Scientific and Ethics Review Unit (12/3823) and the Tufts Health Sciences Institutional Review Board (13205). Additionally, a research permit was granted by the Kenyan National Commission for Science, Technology, and Innovation.

Note that full information on the approval of the study protocol must also be provided in the manuscript.

Plants

Seed stocks	NA
Novel plant genotypes	NA
Authentication	NA
